# Involvement of TLR7 MyD88-dependent signaling pathway in the pathogenesis of adult-onset Still's disease

**DOI:** 10.1186/ar4193

**Published:** 2013-03-04

**Authors:** Der-Yuan Chen, Chi-Chen Lin, Yi-Ming Chen, Joung-Liang Lan, Wei-Ting Hung, Hsin-Hua Chen, Kuo-Lung Lai, Chia-Wei Hsieh

**Affiliations:** 1 Faculty of Medicine, National Yang-Ming University, No. 155, Sec. 2, Li-Nong Street, Taipei 112, Taiwan; 2 Division of Allergy, Immunology and Rheumatology, Taichung Veterans General Hospital, No. 160, Section 3, Taichung-Kang Road, Taichung, 407, Taiwan; 3 Infection and Immunity Research Center, National Yang-Ming University, No. 155, Sec. 2, Li-Nong Street, Taipei 112, Taiwan; 4 Institute of Microbiology and Immunology, Chung-Shan Medical University, No.110, Sec.1, Jianguo N.Rd., Taichung, 402, Taiwan; 5 Institute of Biomedical Science, National Chung-Hsing University, No.250, Kuo-Kuang Rd., Taichung, 402, Taiwan; 6 Division of Immunology and Rheumatology, China Medical University Hospital, No. 2, Yu-De Rd., Taichung, 404, Taiwan

## Abstract

**Introduction:**

The objective of this study was to investigate the potential role of the Toll-like receptor 7 (TLR7) signaling pathway in the pathogenesis of adult-onset Still's disease (AOSD).

**Methods:**

Frequencies of TLR7-expressing precursor of myeloid dendritic cells (pre-mDCs) and mDCs in 28 AOSD patients, 28 patients with systemic lupus erythematosus (SLE) and 12 healthy controls (HC) were determined by flow cytometry analysis. Transcript and protein levels of TLR7 signaling molecules in peripheral blood mononuclear cells (PBMCs) were evaluated by quantitative PCR and western blotting respectively. Serum cytokines levels were measured by ELISA.

**Results:**

Significantly higher median frequencies of TLR7-expressing pre-mDCs and mDCs were observed in AOSD patients (65.5% and 14.9%, respectively) and in SLE patients (60.3% and 14.4%, respectively) than in HC (42.8% and 8.8%, respectively; both *P *<0.001). Transcript and protein levels of TLR7-signaling molecules, including MyD88, TRAF6, IRAK4 and IFN-α, were upregulated in AOSD patients and SLE patients compared with those in HC. Disease activity scores were positively correlated with the frequencies of TLR7-expressing mDCs and expression levels of TLR7 signaling molecules in both AOSD and SLE patients. TLR7 ligand (imiquimod) stimulation of PBMCs resulted in significantly enhanced levels of interleukin (IL)-1β, IL-6, IL-18 and IFN-α in AOSD and SLE patients. Frequencies of TLR7-expressing mDCs and expression levels of TLR7 signaling molecules significantly decreased after effective therapy.

**Conclusions:**

Elevated levels of TLR7 signaling molecules and their positive correlation with disease activity in AOSD patients suggest involvement of the TLR7 signaling pathway in the pathogenesis of this disease. The overexpression of TLR7 MyD88-dependent signaling molecules may be a common pathogenic mechanism for both AOSD and SLE.

## Introduction

Toll-like receptors (TLRs) represent an important link between innate and adaptive immune responses [1,2]. Recent studies have shown that recognition of self-nucleic acid by TLRs plays a critical role in the pathogenesis of autoimmunity and inflammation [3,4]. TLR expression patterns vary among antigen-presenting cells. For example, human myeloid dendritic cells (mDCs) lack TLR9 but express TLR7, which recognizes nucleic acids [5,6]. Endosomally located TLRs of DCs, such as TLR7, are involved in the tissue inflammation of autoimmune diseases, such as systemic lupus erythematosus (SLE) [3,4,7,8]. Treatment of lupus-prone mice with a dual inhibitor of TLR7 and TLR9 leads to the reduction of autoantibody production and disease activity [9]. Therefore, TLR7-mediated DCs are implicated in the pathogenesis of systemic inflammatory diseases.

TLR7 ligation may induce signal transduction via the myeloid differentiation primary-response protein 88 (MyD88), a common adaptor molecule [2,10,11]. The activation of MyD88 signaling leads to the production of type I IFN and proinflammatory cytokines through a group of cytosolic adaptor molecules, including IL-1 receptor-associated kinase (IRAK)-1/4, tumor necrosis factor receptor- associated factor (TRAF)-6, and IFN regulatory factor (IRF)-5/7 [2,10]. In addition, Thibault *et al*. indicated a critical link between the type 1 IFN pathway and the regulation of TLR7-specific immune responses in a murine SLE model [12].

Adult-onset Still's disease (AOSD) is an inflammatory disorder, characterized by fever, rash, arthritis, involvement of various organs, neutrophilic leukocytosis, and increased acute phase reactants [13,14]. Although aetiopathogenesis of AOSD remains unclear, the interplay of viral infections, genetic factors, and immune dysregulation, including cytokine-mediated inflammation and elevated apoptosis, may contribute to the development of this disease [15-19]. Nucleic acids derived from viruses or released from damaged host cells can act as ligands for TLR7 [2,5,9,20] and may promote inflammatory diseases [9]. Previous studies showed that TLR7 ligation could promote the recruitment of neutrophils and amplification of Th17-driven inflammatory responses in inflammatory disease [21], and TLR7 ligation-generated inflammatory cytokines that combine to potentiate Th17 differentiation [22]. Our recent study also showed an important role of Th17 cells in AOSD pathogenesis [23]. Therefore, we hypothesize that TLR7 plays a potential role in AOSD pathogenesis. However, there are no data concerning the TLR7 signaling pathway in AOSD.

In this study, the expression levels of TLR7 were quantified in circulating precursors of mDCs (pre-mDCs) and in mDCs using flow cytometry analysis. The transcript and protein levels of TLR7 signaling molecules in peripheral blood mononuclear cells (PBMCs) were determined using quantitative PCR and western blotting respectively. We enrolled SLE patients, who have some shared clinical manifestations with AOSD, as the disease control because previous studies have documented TLR7 expression in SLE [9,24,25]. The association of TLR7 expression levels with disease activity parameters and downstream cytokines levels was also investigated. To explore the functional role of TLR7, PBMCs were stimulated with TLR7 ligand, after which, supernatant levels of downstream cytokines were evaluated. The changes in the expression levels of TLR7 signaling molecules during longitudinal follow-up of AOSD patients were also studied.

## Methods

### Patients

Twenty-eight patients with active untreated AOSD (twenty female and eight male patients, mean age ± SD, 37.4 ± 14.8 years) fulfilling the Yamaguchi criteria [26] were enrolled. Patients with infections, malignancies or other rheumatic diseases were excluded. The disease activity for each AOSD patient was assessed using a modified Pouchot score described by Rau *et al*. [27]. After initial investigation for TLR7 signaling, all AOSD patients received corticosteroids and non-steroidal anti-inflammatory drugs (NSAIDs). The disease-modifying anti-rheumatic drugs (DMARDs) used were methotrexate (twenty-three patients), hydroxychloroquine (twenty patients), sulfasalazine (ten patients), and azathioprine (six patients). Twenty-eight age-matched patients (twenty-five female and three male patients, mean age 38.2 ± 8.7 years) fulfilling the 1997 revised criteria of the American College of Rheumatology (ACR) for SLE [28] were included as disease controls for systemic inflammation. Disease activity in SLE was determined by calculating the SLE disease activity index (SLEDAI) [29]. Twelve age-matched healthy volunteers (eight female and four male patients, mean age 36.4 ± 9.2 years), who had no rheumatic disease, were used as normal controls. The Ethics Committee of Clinical Research, Taichung Veterans General Hospital, approved this study and the participant's written consent was obtained according to the Declaration of Helsinki.

### Quantitation of the expression levels of TLR7 in mDCs using flow cytometry analysis

PBMCs were isolated from peripheral blood using Ficoll-Hypaque (Amersham Biosciences, Sweden) density gradient centrifugation. For detection of intracellular expression of TLR7 on pre-mDCs (phenotypically defined as CD14^+^CD11c^+ ^cells), and mDCs (phenotypically defined as CD14^+^CD11c^+ ^cells), we performed flow cytometry analysis for three-colored staining of PBMCs with a mixture of phycoerythrin-Cyanin 5 (PE-Cy5)-conjugated anti-CD14 (Beckman Coulter, Brea, CA, USA), fluorescein isothiocyanate (FITC)-conjugated anti-CD11c (eBioscience, San Diego, CA, USA), and PE-conjugated TLR7-specific mAb (R&D Systems, Minneapolis, MN, USA) using a mild modification of the described technique [24,25]. After staining, the cells were washed and immediately analyzed using flow cytometry (Beckman Coulter). An isotype control IgG1-PE (eBiosciences) was used for TLR7 staining at room temperature in the dark. Data were obtained using an Epics XL (Beckman Coulter), and the results were analyzed using EXPO32 software (Beckman Coulter).

### Determination of the mRNA expression (transcript) levels of TLR7signaling on PBMCs using quantitative PCR (qPCR)

To explore TLR7 signaling in the pathophysiology of AOSD and SLE, we examined the transcript levels for signaling molecules on PBMCs using qPCR. Total cellular RNA was obtained from PBMCs by the guanidinium isothiocyanate method [30] and was quantified by spectrophotometry at 260 nm. A 2.5-μg RNA aliquot was reverse-transcribed with 200 U of Moloney murine leukemia virus reverse transcriptase (Fermentas, Thermo Fisher Scientific Inc. Waltham, MA, USA) according to standard procedures. The qPCR was performed using IQ^2 ^Fast qPCR System (Bio-genesis Technology Inc., Taipei, Taiwan) with a method modified from previous reports [25]. The following oligonucleotide primers for each molecule of TLR7 signaling were designed and synthesized. For *TLR7*, sense primer 5'-TTACCTGGATGGAAACCAGCTACT- 3'and antisense primer 5'-TCAAGGCTGAGAAGCTGTAAGCTA-3'; for MyD88, sense primer 5'-GAGCGTTTCGATGCCTTCAT-3' and antisense primer 5'-CGG ATCATCTCCTGCACAAA-3'; for TRAF6, sense primer 5'-GATGCAGAGGAA TCACTTGGC-3' and antisense primer 5'-GGTCTGTCTTACAAGGCGAC-3'; for IRAK*4*, sense primer 5'-GCTGCTGCAAGAGATGACAG-3' and antisense primer 5'-CACTGTCCTGCAACAGCCTA-3'; for IRF5, sense primer 5'-GGAGCATTT TCTCAATGAGCTCATC-3' and antisense primer 5'-CTGCTACAGGCACCAC CTGTACAGT-3'; for *IFN-α*, sense primer 5'-TGCTTTACTGATGGTCCTGGT-3' and antisense primer 5'-TCATGTCTGTCCATCAGACAG-3', and for the housekeeping gene Glyceraldehyde-3-phosphate dehydrogenase (GAPDH), sense primer 5'-GACCTGACCTGCCGTCTAGAAA-3' and antisense primer 5'-CCTGCTTCACCACCTTCTTGA-3'. PCR was performed in a total volume of 10.0 μL containing 10 ng of cDNA, 5 μL 2 × IQ2 fast qPCR system master mix, 0.375 μL each of oligonucleotide primer, and RNase-free water. Amplification cycles were 95°C for 10 min, followed by 40 cycles of denaturation at 95°C for 10s, then annealing and extension at 60 to 62°C for 30s. To standardize mRNA levels of each target gene, transcript levels of the housekeeping gene *GAPDH *were determined in parallel for each sample. The relative expression level of each target gene was calculated with the comparative threshold cycle (Ct) method and evaluated by:

$${\text{2}}^{-\text{▵▵}\text{Ct}},\text{▵▵}\text{Ct }=\text{ Patient }\left(\text{Ct}{\;}_{\text{target gene}}-\text{Ct}{\;}_{\text{GAPDH}}\right)\;-\text{ Mean of controls }\left(\text{Ct}{\;}_{\text{target gene}}-\text{Ct}{\;}_{\text{GAPDH}}\right).$$

### Determination of protein expression levels of TLR7signaling molecules in PBMCs using western blot analysis

Immunoblot analysis of protein expression of TLR7 signaling molecules in the lysates of PBMCs from AOSD patients, SLE patients, and healthy volunteers were performed as described in our recent study [31]. An equal amount of cell extracts from each set of experiments were fractionated on 6 to 8% SDS-PAGE in running buffer (25mM Tris, 192mM glycine, 0.1% SDS). The gel was run at 90V for 30 minutes then at 130V until the blue dye front reached the bottom. The gel was transferred to polyvinylidene difluoride membrane (PVDF) in transfer buffer (50 mM Tris, 384 mM glycine, 20% methanol) at 21V for 1 hr with the Trans-Blot SD Semi-Dry Electrophoretic Transfer Cell (BIO-RAD, Hercules, CA, USA). The membranes were blocked with 5% BSA in 150 mM NaCl, 20 mM Tris-HCl (pH 7.4), 0.1% Tween-20 (TBST) at room temperature for 1 hr then probed with antibodies for TLR7 signaling molecules (Santa Cruz Biotechnology, Santa Cruz, CA, USA), which was generated by immunizing rabbits with the appropriate peptide and antibodies for β-actin (Santa Cruz Biotechnology) at 4°Covernight. The membranes were washed about three times with TBST, followed by incubation with peroxidase-conjugated secondary antibody (1:6000) at room temperature for 1 hr. The membranes of antibody reaction were washed three times with TBST and performed using the enhanced Immobilon Western Chemiluminescent HRP Substrate (WBKLS0500, Millipore, Billerica, MA, USA) then exposed with the new MegaCam 810 scientific grade CCD camera (UVP, LLC, Upland, CA, USA). The relative expression level of TLR7 signaling molecules was normalized to β-actin, and values were expressed relative to control.

### Determination of serum levels of proinflammatory cytokines

Serum levels of IL-1β, IL-6, IL-18, TNF-α, and IFN-α were determined in AOSD patients, SLE patients and healthy controls using ELISA according to the manufacturer's instructions (eBiosciences).

### *Ex vivo *induction of cytokines on PBMCs treated with TLR7 ligand

To explore the functional role of TLR7 activation in the pathophysiology of AOSD and SLE, we examined the changes in supernatant levels of downstream cytokines on PBMCs treated with TLR7 ligand with a method modified from previous reports [32]. PBMCs were obtained from ten AOSD patients, ten SLE patients, and six healthy controls, and were re-suspended in Roswell Park Memorial Institute (RPMI) 1640 medium (Sigma, USA) supplemented with 100 units/mL penicillin, 100 μg/mL streptomycin, and 10% fetal blood serum in a final concentration of 1 × 10^6 ^cells/well. PBMC samples were incubated at 37°C in a 5% CO_2 _humidified atmosphere for 24 hr in the absence or presence of the TLR7 ligand, imiquimod (5 μg/mL, InvivoGen, San Diego, CA, USA). The cell-free supernatant was harvested, and the levels of TLR7-signaling downstream cytokines, including IL-1β, IL-6, IL-18, TNF-α, and IFN-α were determined by ELISA (eBiosciences). A sample with undetectable cytokines was arbitrarily defined as 0 pg/mL.

### Statistical analysis

Results are presented as the mean ± SD or median (IQR). The nonparametric Kruskal-Wallis test was used for between-group comparison of the frequencies of TLR7-expressing pre-mDCs and mDCs, transcript and protein levels of TLR7 signaling molecules, and serum levels of proinflammatory cytokines. When this test showed significant differences, then the exact *P*-values were determined using the Mann-Whitney *U*-test. The correlation coefficient was obtained by the nonparametric Spearman's rank correlation test. The Wilcoxon signed rank test was employed to compare changes in supernatant cytokines levels from TLR7 ligand-treated PBMCs, and changes in transcript levels of TLR7 signaling molecules during follow-up in AOSD patients after effective therapy. Probability less than 0.05 was considered significant.

## Results

### Clinical characteristics of AOSD patients and SLE patients

As illustrated in Table 1, all patients with active AOSD had spiking fevers (≥39°C). Evanescent rash, arthritis, sore throat, and lymphadenopathy were noted in 25 (89.3%), 20 (71.4%), 18 (64.3%), and 7 (25.0%) patients respectively. All SLE patients had active disease (mean SLEDAI ± SD, 7.8 ± 2.3, range 6.0 to 14.0) at the time of investigation and nine patients (32.1%) had renal involvement. However, there were no significant differences between AOSD patients and SLE patients in age at onset, proportion of females, or frequencies of extra-renal manifestations.

**Table 1:** Demographic data and clinical characteristics of patients with adult-onset Still's disease (AOSD), patients with systemic lupus erythematosus (SLE) and healthy controls (HC)

Characteristics	AOSD (*n *= 28)	SLE (*n *= 28)	HC (*n *= 12)
Age at study entry, years	37.4 ± 14.8	38.2 ± 8.7	36.4 ± 9.2
Proportion of females	20 (71.4%)	25 (89.3%)	8 (66.7%)
Fever (≧39°C)	28 (100%)	24 (85.8%)	NA
Rash	25 (89.3%)	21 (75.0%)	NA
Arthritis	20 (71.4%)	15 (53.6%)	NA
Lymphadenopathy	7 (25.0%)	6 (21.4%)	NA
Nephritis	0 (0.0%)	9 (32.1%)*	NA
Ferritin levels, μg/L	931.0 ± 106.5	NA	NA
AOSD activity score	5.93 ± 1.46	NA	NA
SLEDAI	N A	7.8 ± 2.3	NA
C3 levels, mg/dL	NA	84.6 ± 24.7	NA
C4 levels, mg/dL	NA	19.5 ± 8.9	NA
Anti-ds DNA, U/mL	NA	183.2 ± 200.3	NA

Data are presented as mean ± SD or number (percentage); NA, not applicable. Nephritis is defined by persistent proteinuria (>0.5 g/24 hours), the presence of cellular casts, or pathological examination of renal biopsy specimens showing lupus nephritis. C3 and C4, complement 3 and 4; Anti-ds DNA, anti-double strand DNA antibody; SLEDAI, SLE disease activity index; *P <0.001, versus AOSD patients.

### The circulating levels of TLR7-expressing mDCs in AOSD patients and SLE patients

**Figure 1:**
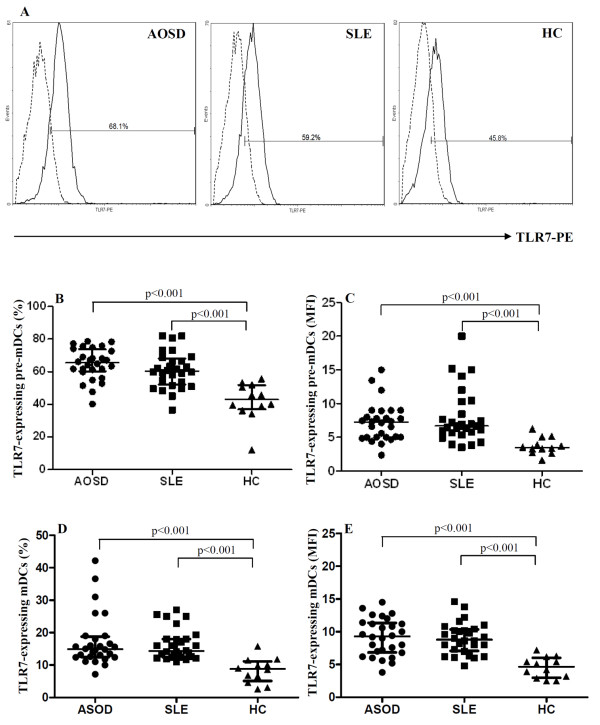
**TLR7 expression on circulating precursor myeloid dendritic cells (pre-mDCs) and mDCs in patients with adult-onset Still's disease (AOSD), patients with systemic lupus erythematosus (SLE), and healthy volunteers**. Representative examples (**A**) of flow cytometric histograms of Toll-like receptor (TLR)7 expression in mDcs (mDCs) obtained from peripheral blood of one representative patient with active AOSD, a patient with SLE and a healthy control (HC). The frequencies and mean fluorescence intensity (MFI) of TLR7 expression on circulating pre-mDCs [CD14^+^CD11c^+ ^cells] (**B **and **C**, respectively) and mDCs [CD14^-^CD11c^+ ^cells] (D and E, respectively) were obtained from 28 patients with active AOSD, 28 with active patients, and 12 HCs. The horizontal line indicates the median value for each group. The *P*-value was determined by the Mann-Whitney *U*-test.

Because the classical CD14+ monocytes constitute the vast majority of all monocytes in peripheral blood, we examined the percentages of TLR7-expressing cells in each subset of myeloid DCs, including pre-mDCs (CD14^+^CD11c^+ ^cells) and mDCs (CD14^-^CD11c^+ ^cells). As shown in Figure 1, significantly higher percentages of TLR7-expressing pre-mDCs and mDCs were observed in patients with active AOSD (median 65.5%, IQR 60.2 to 73.7%, and 14.9%, IQR 12.5 to 18.8%; for pre-mDCs and mDCs, respectively) and SLE (median 60.3%, IQR 52.1 to 68.1% and 14.4%, IQR 12.5 to 18.0%, respectively) than in healthy controls (median 42.8%, IQR 36.9 to 51.7% and 8.8%, IQR 5.1 to 11.1%, respectively) (all *P *<0.001). Significantly higher mean fluorescence intensity (MFI) of TLR7 staining on circulating pre-mDCs and mDCs was also observed in patients with active AOSD (median 7.23, IQR 5.00 to 8.69 and 9.30, IQR 6.85 to 11.35, respectively) and in active SLE (median 6.69, IQR 5.69 to 9.84 and 8.80, IQR 7.08 to 10.35, respectively) than in healthy controls (median 3.47, IQR 2.88 to 4.78 and 4.65, IQR 2.98 to 6.00, respectively) (all *P *<0.001). However, there was no significant difference between AOSD patients and SLE patients in the expression level of TLR7 on circulating pre-mDCs or mDCs. There was no significant difference between the two patient groups and the healthy controls in the numbers of pre-mDCs or mDCs cells that were gated for MFI (data not shown).

### The transcript and protein levels of TLR7 MyD88-dependent signaling pathway

**Figure 2:**
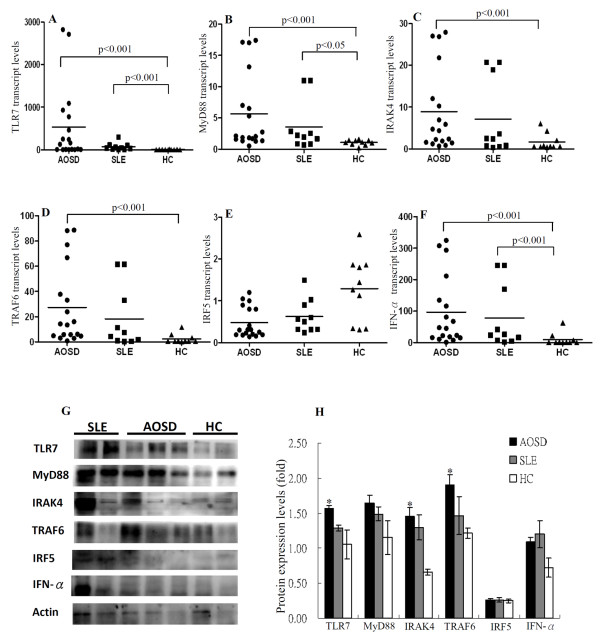
**Transcript and protein levels of Toll-like receptor (TLR)7 signaling molecules in patients with adult-onset Still's disease (AOSD), patients with systemic lupus erythematosus (SLE), and healthy volunteers**. The comparison in transcript levels of TLR7 signaling molecules, including TLR7 (**A**), Myeloid differentiation primary-response protein 88 (MyD88) (**B**), IL-1 receptor-associated kinase (IRAK)4 (**C**), TNF receptor-associated factor (TRAF)6 (**D**), IFN regulatory factor (IRF)5 (**E**), and IFN-α (**F**) among AOSD patients, SLE patients, and healthy controls (HCs). The horizontal line indicates median value for each group. Immunoblot analyses (**G**) for protein expression of TLR7 signaling molecules in the lysates of peripheral blood samples from six AOSD patients, three SLE patients and three healthy volunteers. The comparison in the protein expression levels of TLR7 signaling molecules among three groups (**H**). Bars show the mean ± standard error of the mean; *P <0.05 versus healthy controls (Mann-Whitney *U*-test).

As shown in Figure 2, significantly higher transcript levels of TLR7, MyD88, IRAK4, TRAF6, and IFN-α were observed in active AOSD patients (median 145.64, IQR 7.07 to 814.81; 2.08, IQR 1.53 to 8.55; 4.55, IQR 1.55 to 14.49; 13.86, IQR 4.80 to 45.04; 46.23, IQR 15.51 to 154.05, respectively) than those in healthy controls (0.48, IQR 0.23 to 8.62; 1.12, IQR 0.79 to 1.40; 0.59, IQR 0.51 to 2.62; 0.55, IQR 0.40 to 3.80; 0.61, IQR 0.10 to 11.30, respectively (all *P *<0.001). Significantly higher transcript levels of TLR7, MyD88, and IFN-α were also observed in active SLE patients (median 43.47, IQR 9.30 to 103.07; 2.08, IQR 0.85 to 4.87; 24.91, IQR 6.20 to 188.90, respectively) than those in healthy controls (*P *<0.001, *P *<0.05, *P *<0.001, respectively). No significant differences were observed between AOSD or SLE patients and healthy controls in the transcript levels of IRF-5.

As illustrated in Figure 2G and 2H, protein expression levels of TLR7, MyD88, IRAK4, TRAF6, and IFN-α were upregulated in patients with active AOSD (relative expression levels, mean ± SEM, 1.57 ± 0.04, 1.65 ± 0.12, 1.45 ± 0.13, 1.91 ± 0.14, 1.09 ± 0.06, respectively) and SLE (1.29 ± 0.04, 1.49 ± 0.10, 1.30 ± 0.18, 1.47 ± 0.27, 1.20 ± 0.20; respectively) compared to healthy controls (1.05 ± 0.20, 1.15 ± 0.24, 0.66 ± 0.04, 1.21 ± 0.07, 0.72 ± 0.14, respectively). No significant difference was observed between AOSD or SLE patients and healthy controls in the expression levels of IRF-5 protein. Elevated levels of TLR7 expression co-existed with the expression levels of MyD88-dependent signaling molecules. These data suggested an activation of TLR7 signaling pathway in both AOSD patients and SLE patients.

### Serum levels of proinflammatory cytokines and IFN-α

**Figure 3:**
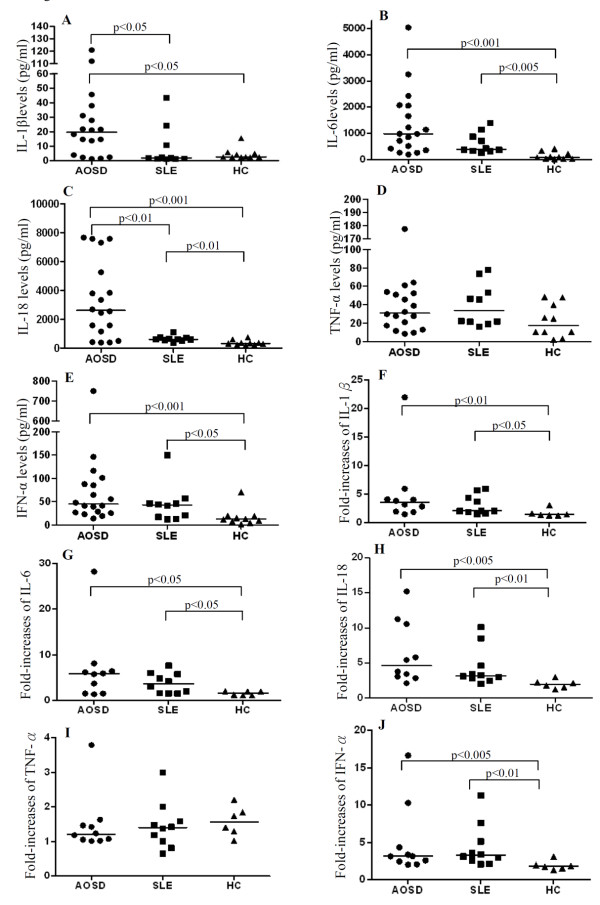
**Serum cytokines levels and the fold-increases of supernatant cytokines levels after stimulation with the Toll-like receptor (TLR)7 ligand**. The comparison in serum levels of cytokines, including IL-1β (**A**), IL-6 (**B**), IL-18 (**C**), TNF-α (**D**), and IFN-α (**E**) among patients with adult-onset Still's disease (AOSD), patients with systemic lupus erythematosus (SLE), and healthy controls (HCs). To explore the functional role of TLR7, we examined the fold-increases of supernatant cytokines levels, including IL-1β (**F**), IL-6 (**G**), IL-18 (**H**), TNF-α (**I**), and IFN-α (**J**) after stimulation with the TLR7 ligand (imiquimod 5 μg/mL) in AOSD patients, SLE patients, and HCs. The horizontal line indicates the median value for each group. The *P*-value was determined by the Mann-Whitney *U*-test.

As shown in Figure 3A-E, significantly higher median levels of serum IL-1β, IL-6, IL-18, and IFN-α in patients with active AOSD (19.65 pg/mL, IQR 3.55 to 32.79 pg/mL, 984.98 pg/mL, IQR 409.24 to 2064.92 pg/mL, 2,613.44 pg/mL, IQR 983.04 to 5,765.09 pg/ml, and 45.02 pg/mL, IQR 26.27 to 91.26 pg/mL, respectively) and higher median levels of serum IL-6, IL-18, and IFN-α in active SLE patients (405.48 pg/mL, IQR 330.32 to 945.83 pg/mL; 605.10 pg/mL, IQR 539.52 to 708.98 pg/mL, and 43.21 pg/mL, IQR 16.23 to 49.12 pg/mL, respectively) were observed when compared to those in healthy controls (2.61 pg/mL, IQR 2.49 to 5.22 pg/mL, *P *<0.05 for IL-1β; 85.78 pg/mL, IQR 33.38 to 249.22 pg/mL, *P *<0.001 and *P *<0.005 for IL-6; 322.69 pg/mL, IQR 222.50 to 424.03pg/mL, *P *<0.001 and *P *<0.01 for IL-18, and 12.79 pg/mL, IQR 6.56 to 19.34pg/mL, *P *<0.001 and *P *<0.05 for IFN-α). However, there were no significant differences between AOSD or SLE patients and healthy controls in serum levels of TNF-α.

### Correlation between disease activity and circulating levels of TLR7-expressing mDCs or the transcript levels for TLR7-signaling molecules

The disease activity scores were positively correlated with the percentages of TLR7-expressing pre-mDCs and mDCs in AOSD patients (*r *= 0.600 and *r *= 0.597, respectively, both *P *<0.005) and SLE patients (*r *= 0.477 and *r *= 0.422, respectively, both *P *<0.05). As illustrated in Table 2, the disease activity scores were significantly correlated with transcript levels for TLR7 and MyD88-dependent signaling molecules, including MyD88, TRAF6, and IFN-α in AOSD patients. Similarly, SLEDAI scores were positively correlated with transcript levels of TLR7, MyD88, and IFN-α in SLE patients. In addition, transcript levels of TLR7 were positively correlated with expression levels of MyD88-dependent signaling molecules, including MyD88, TRAF6, IRAK4, and IFN-α in AOSD patients. The transcript levels of TLR7 and MyD88-dependent signaling molecules were positively correlated with serum levels of IL-1β, IL-6, IL-18, and IFN-α in AOSD patients (Table 2).

**Table 2:** Correlation between transcript levels for TLR7 signaling molecules, and disease activity score and serum cytokine levels in AOSD and SLE patients

AOSD patients	TLR7	MyD88	TRAF6	IRAK4	IRF5	IFN-α
Activity score	0.766***	0.493*	0.481*	0.488*	0.151	0.489*
MyD88 transcript	0.818***	--				
TRAF6 transcript	0.742***	0.789***	--			
IRAK4 transcript	0.701**	0.746***	0.986***	--		
IRF5 transcript	0.241	0.223	0.231	0.218	--	
IFN-α transcript	0.713**	0.738***	0.988***	0.986***	0.254	--
Serum IL-1β level	0.626**	0.424	0.589*	0.531*	-0.131	0.556*
Serum IL-6 level	0.589*	0.571*	0.476*	0.434	-0.131	0.455
Serum IL-18 level	0.732**	0.531*	0.451	0.459	-0.033	0.408
Serum TNF-α level	0.117	0.030	0.104	0.059	-0.094	0.036
Serum IFN-α level	0.482*	0.517*	0.558*	0.589*	-0.429	0.571*

**SLE **patients	TLR7	MyD88	TRAF6	IRAK4	IRF5	IFN-α

SLEDAI score	0.782**	0.731*	0.486	0.517	0.355	0.698*
MyD88 transcript	0.806**	--				
TRAF6 transcript	0.564	0.842***	--			
IRAK4 transcript	0.515	0.685*	0.939***	--		
IRF5 transcript	0.115	-0.236	-0.152	0.127	--	
IFN-α transcript	0.661*	0.855***	0.976***	0.952***	-0.139	--
Serum IL-1β level	0.661*	0.334	0.298	0.225	-0.103	0.274
Serum IL-6 level	0.624	0.134	0.036	0.012	-0.024	0.036
Serum IL-18 level	0.770**	0.770**	0.648*	0.588	-0.055	0.721*
Serum TNF-α level	0.261	0.219	0.413	0.547	-0.292	0.474
Serum IFN-α level	0.212	0.456	0.359	0.444	-0.395	0.298

AOSD, adult-onset Still's disease; SLE, systemic lupus erythematosus; SLEDAI, SLE disease activity index; MyD88, Myeloid differentiation primary-response protein 88; TRAF6, TNF receptor associated factor-6; IRAK4, IL-1 receptor-associated kinase-4; IRF5, interferon regulatory factor-5. *P <0.05, **P <0.01, ***P <0.001 (Spearman's rank correlation test).

### Functionality of TLR7-mediated production of proinflammatory cytokines and IFN-α

We examined whether this enhanced TLR7 expression was functional in terms of cytokine production. Changes in supernatant levels of TLR7-signaling downstream cytokines on PBMCs treated with TLR7-ligand (imiquimod, 5 μg/mL) in ten AOSD patients, ten SLE patients and six healthy volunteers were analyzed. Our results showed that TLR7 ligand stimulation of PBMCs from patients with AOSD and SLE induced greater-fold increases of IL-1β (median, 3.5, range 1.5 to 22.0, *P *<0.01 and 2.1, range 1.5 to 6.0, *P *<0.05, respectively), IL-6 (median, 5.9, range 1.4 to 28.2 and 3.6, range 1.5 to 7.6, respectively, both *P *<0.05), IL-18 (median, 4.6, range 3.0 to 10.7, *P *<0.005 and 3.2, range 2.7 to 5.6, *P *<0.01, respectively), and IFN-α (median, 3.1, range 2.0 to 16.6, *P *<0.005 and 3.2, range 2.1 to 11.3, *P *<0.01, respectively) compared with PBMCs from healthy controls (median, 1.4, range 1.3 to 3.1; 1.6, range 1.1 to 2.0; 2.0, range 1.5 to 2.4; and 1.8, range 1.3 to 3.1, respectively, Figure 3F-H). However, imiquimod stimulation of PBMCs did not result in significant amplification of TNF-α in AOSD patients or SLE patients.

### Changes in expression levels of TLR7 MyD88-dependent signaling molecules in AOSD patients after therapy

**Figure 4:**
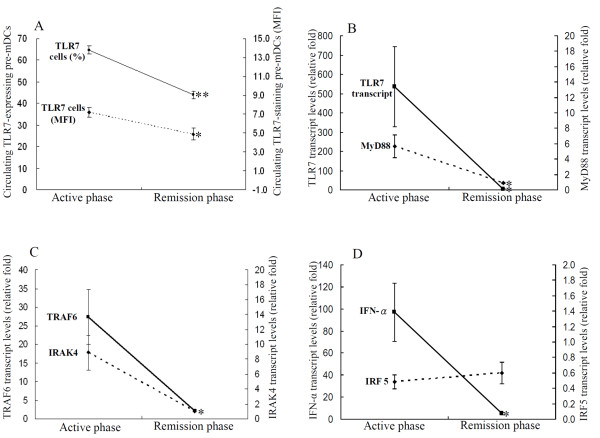
**Change in circulating frequencies of Toll-like receptor (TLR)7-expressing precursor myeloid dendritic cells (pre-mDCs) and transcript levels of TLR7-signaling molecules after therapy**. The changes in the percentages of circulating TLR7-expressing pre-mDCs (**A**) and in the transcript levels of TLR7-signaling molecules (B, **C**, and **D**), including TLR7, Myeloid differentiation primary-response protein 88 (MyD88), IL-1 receptor-associated kinase (IRAK)4, TNF receptor-associated factor (TRAF)6, IFN regulatory factor (IRF)5, and IFN-α in 12 AOSD patients after therapy. Data are presented as mean ± standard error of the mean. *P <0.005, **P <0.001, versus active phase, determined by the Wilcoxon signed rank test.

Eighteen AOSD patients were available for examination of TLR7-signaling expression in both the active phase and the remission phase. As shown in Figure 4A, the percentages and MFI of TLR7-expressing pre-mDCs significantly decreased (mean ± SEM, 64.78 ± 1.83 vs. 43.95 ± 1.82; 7.19 ± 0.54 vs. 4.90 ± 0.61, respectively, both *P *<0.001), paralleling clinical remission and the decrease in serum ferritin levels (931.5 ± 201.3 μg/L vs. 234.4 ± 28.3 μg/L, *P *<0.001) in AOSD patients. Similarly, the transcript levels for TLR7-signaling molecules including TLR7, MyD88, TRAF6, IRAK4, and IFN-α significantly decreased (535.8 ± 207.8 vs. 2.93 ± 2.26, *P *<0.001; 5.66 ± 1.44 vs. 0.92 ± 0.09, *P *<0.001; 27.40 ± 7.33 vs. 2.10 ± 0.35, *P *<0.005; 8.90 ± 2.34 vs. 1.02 ± 0.12, *P *<0.005; 96.94 ± 26.29 vs. 4.99 ± 1.07, *P *<0.001, respectively, Figure 4B, C, and D), paralleling the decrease in disease activity score (6.44 ± 0.40 vs. 3.78 ± 0.15, *P *<0.001) in AOSD patients.

## Discussion

This study is the first attempt to characterize the expression of TLR7 on mDCs and TLR7 MyD88-dependent signaling molecules on PBMCs in AOSD patients. In order to avoid the effects of immunosuppressive agents on our results, new-onset untreated AOSD patients were enrolled. Our results showed significantly elevated frequencies of TLR7-expressing mDCs and upregulated levels of TLR7 transcript and protein on PBMCs. The expression levels of TLR7 were positively correlated with disease activity in AOSD patients. Moreover, a parallel decrease in TLR7 expression levels with disease remission was found in our AOSD patients. Our observations indicate that TLR7 overexpression is involved in the pathogenesis of AOSD. However, a large prospective study should be conducted to confirm our findings.

Similar to AOSD patients, our SLE patients had significantly elevated frequencies of circulating TLR7-expressing mDCs and upregulated levels of TLR7 expression, which were correlated with SLEDAI scores. Our results were consistent with the findings of recent studies showing elevated expression levels of circulating TLR7 transcript using the qPCR method [25], and were similar to the results of recent studies showing a role for TLR7 genes in the predisposition of Asian populations to SLE [33,34]. In addition, Christensen *et al*. revealed that TLR7-deficient lupus-prone mice had ameliorated disease and decreased lymphocyte activation [35]. These findings suggest that TLR7 expression is involved in the pathogenesis of SLE, and elevated frequencies of TLR7-expressing pre-mDCs and mDCs may be a common characteristic of systemic inflammatory diseases including AOSD and SLE.

TLR7 ligation triggers activation of a group of cytosolic adaptor molecules [11]. MyD88 acts as an adaptor that recruits the serine-threonine kinase IRAK and TRAF6 to the TLR7 signaling pathway. MyD88-mediated signaling lies at the center of the TLR-driven immune response [2,11,36], and leads to production of proinflammatory cytokines and type I IFN [10,11,37]. Our results showed elevated transcript and protein levels of TLR7 MyD88-dependent signaling molecules, including MyD88, IRAK4 and TRAF6 on PBMCs from both AOSD patients and SLE patients. Moreover, a positive correlation between disease activity and the expression levels of TLR7 MyD88-dependent signaling molecules were observed in both AOSD patients and SLE patients (Table 2). In concordance with the findings of previous studies showing that TLR7 activation triggers production of proinflammatory cytokines [10,11,38], our results showed elevated levels of serum IL-1β, IL-6, IL-18, and IFN-α positively correlated with the expression levels of TLR7 signaling molecules in AOSD patients. These observations suggest the pathogenic role of the TLR7 MyD88-dependent signaling pathway in AOSD. Although IRF5 is important for regulation of IFN-α after TLR activation [39], the absence of a significant increase in IRF5 expression in our patients may be related to the enrolled patients' characteristics, differences in experimental procedures and/or the small sample size in our study.

Accumulating evidence shows that IFN-α, a type I IFN, plays a pivotal role in triggering and sustaining inflammatory diseases [40,41]. Previous studies have identified type I IFN gene expression in PBMCs from patients with active lupus [42,43], and overproduction of IFN-α, which correlates with disease exacerbation in SLE [43,44]. Although there are no data on IFN-α in AOSD, elevated levels of IFN-α, which correlated with disease activity in our AOSD patients, suggest the potential role of IFN-α in AOSD pathogenesis. Moreover, we observed a positive correlation between expression levels of TLR7-signaling molecules and IFN-α level in AOSD patients and SLE patients, consistent with the findings of a previous study showing the concordant overexpression of TLR7 and IFN-α in SLE patients [25] and IFN-α production requiring TLR7/MyD88 signaling in experimental mouse lupus [45]. Our results support the observation that TLR7 inhibitors have a therapeutic application in autoimmune dermatitis with a prominent IFN-α signature [46].

Given a positive association of TLR7 expression with levels of proinflammatory cytokines, we further investigated the functional relation between TLR7 ligation and the downstream mediators. Our results showed that TLR7 ligand (imiquimod) stimulation of PBMCs induced greater-fold increases in IL-1β levels (up to around 22-fold), IL-6 (up to around 28-fold), IL-18 (up to around15-fold), and IFN-α (up to around 17-fold) in AOSD patients compared to those in healthy controls, indicating that the upregulation of TLR7 is functional. Moreover, we revealed that the cytokine pattern induced by TLR7 ligand stimulation overlaps with a similar serum cytokine panel observed in both AOSD and SLE (Figure 3), suggesting that TLR7 triggering has an important contribution to the inflammatory response in both diseases. Our results were consistent with the findings of a study showing that imiquimod induced production of proinflammatory cytokines and IFN-α [32]. Our data also showed enhanced production of IL-18 after TLR7 ligand stimulation, supporting the findings of sharing of the TLR7 with IL-18 receptor signaling [2,47]. However, our results are different from the findings of a recent study showing no significant difference in the induction of all measured cytokines between SLE patients and controls [24]. This discrepancy may be related to the difference in the TLR7 ligand used (R837 vs. imiquimod in our study) and in disease activity in SLE patients in our study (SLEDAI, mean ± SD, 3.1 ± 3.0 vs. 7.8 ± 2.3).

Our longitudinal follow-up of AOSD patients showed that the expression levels of TLR7 MyD88-dependent signaling molecules, including TLR7, MyD88, IRAK4, TRAF6, and IFN-α, decreased significantly, paralleling the clinical remission and a decrease in inflammatory parameters after therapy (Figure 4). Our results support the hypothesis that inhibitors of TLR7 signaling and anti-IFN-α therapy, can be a promising therapeutic modality for systemic inflammatory diseases [9,48-51].

There were some limitations in our study. Because it was difficult to obtain biopsy tissue, we could not investigate the expression of TLR7 signaling molecules on lesion specimens in AOSD patients. To prove that TLR7 signaling is active in AOSD *in vivo*, further study to investigate phosphorylation of signaling molecules in freshly isolated cells is needed. The lack of significant correlations between expression levels of TLR7 signaling and clinical features of AOSD may be due to the small sample size in this clinically heterogeneous disease. In addition, it is likely that more than one TLR pathway is needed for the initiation of inflammatory response in AOSD. Therefore, a previous investigation suggested that simultaneous or sequential triggering of different TLR pathways is needed to develop an inflammatory disease [52].

## Conclusions

Our results show that TLR7 activation with increased production of proinflammatory cytokines and IFN-α through MyD88-dependent signaling may be involved in the pathogenesis of both AOSD and SLE. We also provide the first evidence that IFN-α overexpression may have a possible link with immune response in AOSD. Such studies are of translational and fundamental interest, because they can provide potentially therapeutic modalities [48-51], and may shed light on the etiopathogenesis of the TLR7 signaling pathway in systemic inflammatory diseases. Further study on the pathobiology of the TLR7 MyD88-dependent signaling pathway in AOSD is needed.

## Abbreviations

ACR: American College of Rheumatology: AOSD: adult-onset Still's disease; BSA: bovine serum albumin; Ct: threshold cycle; DMARD: disease-modifying anti-rheumatic drug; ELISA: enzyme-linked immunosorbent assay; FITC: fluorescein isothiocyanate; GAPDH: glyceraldehyde-3-phosphate dehydrogenase; IFN-α: interferon-α; IL: interleukin; IRAK: IL-1 receptor-associated kinase; IRF: IFN regulatory factor; mAb monoclonal antibody; mDC: myeloid dendritic cell; MFI: mean fluorescence intensity; MyD88: myeloid differentiation primary-response protein 88; NSAID: non-steroidal anti-inflammatory drug; PBMC: peripheral blood mononuclear cell; PCR: polymerase chain reaction; PE-Cy5: phycoerythrin-Cyanin 5; qPCR: quantitative PCR; PVDF: polyvinylidene difluoride membrane; RPMI: Roswell Park Memorial Institute; SLE: systemic lupus erythematosus; SLEDAI: SLE disease activity index; TBST: Tris-buffered saline and Tween 20; TLR: Toll-like receptor; TNF: tumor necrosis factor; TRAF: TNF receptor-associated factor.

## Competing interests

The authors declare that they have no competing interests.

## Authors' contributions

All authors made substantive intellectual contributions to the present study and approved the final manuscript. DYC conceived the study, generated the original hypothesis, designed the study, acquired clinical data, analyzed data, and drafted and revised the manuscript. CCL generated the original hypothesis, designed the study, analyzed data and drafted the manuscript. YMC, WTH, and JLL acquired clinical data and performed statistical analysis. HHC, KLL, and CWH performed clinical assessments on study subjects.
